# Spatiotemporal Developmental Upregulation of Prestin Correlates With the Severity and Location of Cyclodextrin-Induced Outer Hair Cell Loss and Hearing Loss

**DOI:** 10.3389/fcell.2021.643709

**Published:** 2021-05-24

**Authors:** Dalian Ding, Haiyan Jiang, Senthilvelan Manohar, Xiaopeng Liu, Li Li, Guang-Di Chen, Richard Salvi

**Affiliations:** Center for Hearing and Deafness, University at Buffalo, Buffalo, NY, United States

**Keywords:** cyclodextrin, prestin, Niemann-Pick C1, spiral ganglion neurons, outer hair cells, inner hair cells, otoacoustic emissions, compound action potential

## Abstract

2-Hyroxypropyl-beta-cyclodextrin (HPβCD) is being used to treat Niemann-Pick C1, a fatal neurodegenerative disease caused by abnormal cholesterol metabolism. HPβCD slows disease progression, but unfortunately causes severe, rapid onset hearing loss by destroying the outer hair cells (OHC). HPβCD-induced damage is believed to be related to the expression of prestin in OHCs. Because prestin is postnatally upregulated from the cochlear base toward the apex, we hypothesized that HPβCD ototoxicity would spread from the high-frequency base toward the low-frequency apex of the cochlea. Consistent with this hypothesis, cochlear hearing impairments and OHC loss rapidly spread from the high-frequency base toward the low-frequency apex of the cochlea when HPβCD administration shifted from postnatal day 3 (P3) to P28. HPβCD-induced histopathologies were initially confined to the OHCs, but between 4- and 6-weeks post-treatment, there was an unexpected, rapid and massive expansion of the lesion to include most inner hair cells (IHC), pillar cells (PC), peripheral auditory nerve fibers, and spiral ganglion neurons at location where OHCs were missing. The magnitude and spatial extent of HPβCD-induced OHC death was tightly correlated with the postnatal day when HPβCD was administered which coincided with the spatiotemporal upregulation of prestin in OHCs. A second, massive wave of degeneration involving IHCs, PC, auditory nerve fibers and spiral ganglion neurons abruptly emerged 4–6 weeks post-HPβCD treatment. This secondary wave of degeneration combined with the initial OHC loss results in a profound, irreversible hearing loss.

## Introduction

Cyclodextrins are widely used in many industries because they can encapsulate hydrophobic compounds within a hydrophobic shell (Okamura et al., 2002; Laza-Knoerr et al., 2010; Loftsson and Brewster, 2010). Consequently, cyclodextrins are often employed as excipients because they enhance the solubility, absorption, and stability of these compounds (Otero-Espinar et al., 2010), making them extremely useful for drug delivery (Davis and Brewster, 2004; Tang et al., 2006; Ren et al., 2010; Wu et al., 2013). At the doses normally employed commercially, cyclodextrins have negligible side effects (Marttin et al., 1998; Uchenna Agu et al., 2000; Stella and He, 2008) and are considered safe (FDA; Notices 000155, 000074, 000046).

Because cyclodextrins can remove lipids and cholesterol from cells, they are being evaluated as potential therapeutics to treat vascular, kidney, liver, and neurodegenerative diseases caused by the cellular accumulation of cholesterol (Lirussi et al., 2002; Walenbergh et al., 2015; Coisne et al., 2016; Zimmer et al., 2016; Mitrofanova et al., 2018; Bessell et al., 2019; Cho et al., 2019; Kim et al., 2020). 2-Hydroypropyl-beta-cyclodextrin (HPβCD) is currently being evaluated as an investigational new drug to treat Niemann-Pick Type C1 disease (NPC1) (ClinicalTrials.gov Identifier: NCT02534844), a fatal neurodegenerative disease caused by the buildup of unesterified cholesterol in brain (Kolodny, 2000; Ory, 2000; Newton et al., 2018). Unesterified cholesterol plays an important role in plasma membrane turnover, which in neurons, is estimated to be more than 20%/day (Dietschy and Turley, 2004). In the brain, inactivation of NPC1 leads to the sequestration of unesterified cholesterol in endosomes and lysosomes resulting in neurodegeneration (Aqul et al., 2011). Mutant *npc*1^–/–^ mice, an animal model of NPC1 disease, develop severe pulmonary, hepatic and neurodegenerative disorders. However, when *npc1*^–/–^ mice are treated with high systemic doses of HPβCD, the compound overcomes the cholesterol transport deficits allowing the release of excess sterols thereby alleviating symptoms (Liu et al., 2010; Ramirez et al., 2010; Taylor A. M. et al., 2012). Although HPβCD slows NPC1 neurodegeneration, the high therapeutic doses employed can cause hearing loss (Ward et al., 2010; Crumling et al., 2012; King et al., 2014; Cronin et al., 2015; Maarup et al., 2015; Liu et al., 2020). Cyclodextrin-induced hearing loss has an abrupt onset due to the rapid degeneration of outer hair cells (OHC; Crumling et al., 2012, 2017; Cronin et al., 2015; Takahashi et al., 2016). Over time, the inner hair cells (IHCs), spiral ganglion neurons (SGNs), and auditory nerve fibers (ANFs) eventually degenerate following high-dose cyclodextrin treatment, but the time course over which this occurs is unclear (Liu et al., 2020; Ding et al., 2021). Because HPβCD initially destroys only OHCs and because cholesterol modulates OHC electromotility mediated by prestin (Rajagopalan et al., 2007), it was assumed that this selective damage was related to the expression of the electromotile protein prestin located in the plasma membrane along the OHC lateral wall (Takahashi et al., 2016; Crumling et al., 2017). Because mutant mice with a non-electromotile version of prestin continued to be vulnerable to HPβCD ototoxicity; it was concluded that electromotility per se was not a critical factor in HPβCD ototoxicity (Zhou et al., 2018). On the other hand, OHCs in the middle and basal turn of prestin knockout mice were substantially less susceptible to HPβCD toxicity than wild type (WT) mice. Despite the absence of prestin, approximately 20% more OHCs were damaged by HPβCD in the knockout mice compared to saline controls (Takahashi et al., 2016). Moreover, there was still considerable OHC loss in the apical turn of prestin knockout mice. While these results indicate that prestin is important, other factors may contribute to cyclodextrin ototoxicity.

Prestin undergoes a developmental spatial and temporal upregulation in altricial mammals. In rodents, prestin expression in the OHC lateral wall progressively increases from postnatal day 0 (P0) reaching adult-like levels in the basal turn at approximately P9, in the middle turn around P11 and in the apical turn around P12 (Belyantseva et al., 2000; Jensen-Smith and Hallworth, 2007). In contrast, cholesterol expression in the OHC lateral wall decreases slightly from P3 to P21 (Jensen-Smith and Hallworth, 2007). If prestin expression is a key element in cyclodextrin ototoxicity, then high-dose HPβCD should only damage basal turn OHCs if the drug is administered in early postnatal life, but OHC damage should progressively increase and spread from the base to the apex of the cochlea from P0 to adulthood. To test this hypothesis, the developmental expression of prestin was evaluated in OHCs along the length of the cochlea and compared to the cochlear frequency-dependent functional deficits and time-dependent histopathological damage patterns in the cochlea. By carefully assessing the time course of cyclodextrin ototoxicity over several months, we were able to show that HPβCD, in addition to causing early onset OHC damage, also caused a large, secondary wave of damage to IHCs, ANFs and SGNs between 4- and 6-weeks after HPβCD treatment.

## Materials and Methods

### Subjects

Sprague-Dawley rats (Charles River) were used for all experiments. In Experiment #1, the developmental upregulation of prestin was evaluated in the OHCs of postnatal day 5 (P5, *n* = 4), day 10 (P10, *n* = 4), day 15 (P15, *n* = 4) and day 28 (P28, *n* = 4) rats. In Experiments #2, P5 (*n* = 6), P10 (*n* = 6), P15 (*n* = 6), and P28 (*n* = 6) Sprague-Dawley rats were treated subcutaneously with a single dose of 4,000 mg/kg of HPβCD and a Control group (*n* = 10) was treated similarly with saline. The cochleae from these animals underwent histological analysis 8-weeks post-treatment (*n* = 6/group). DPOAEs and CAPs were measured from the P5 (*n* = 6), P10 (*n* = 6), and P15 (*n* = 6) groups 8-weeks after HPβCD treatment. DPOAEs (*n* = 10) and CAPs (*n* = 8) were also measured from the saline Control group. In Experiments #3, which was designed to assess the time-course of degeneration, the extent of hair cell, nerve fiber and SGN loss was evaluated in adult rats (∼12 weeks old at time of HPβCD treatment) as a function of time after treatment with 4,000 mg/kg of HPβCD. Sprague-Dawley rats were evaluated 1-week (*n* = 5) and 4- (*n* = 4), 6- (*n* = 6) or 8-weeks (*n* = 4) post-treatment and the results compared to a saline Control group (*n* = 6) 8-weeks post-treatment.

### HPβCD Treatment

2-Hyroxypropyl-beta-cyclodextrin was administered subcuta-neously to rats at a dose of 4,000 mg/kg of HPβCD in sterile saline (volume: 0.2 ml for P5, P10, P15, and P28 rats and 1 ml for adult rats). This high 4,000 mg/kg dose was used in order to destroy OHCs over the length of the cochlea in order to assess the developmental upregulation of prestin from the base to apex of the cochlea. Rats in the Control group (*n* = 6) were subcutaneously injected with the same amount of sterile saline. One ear of each rat in the experimental group was used to prepare cochlear surface preparations; the other was used to prepare temporal bone sections. Animals were sacrificed for histological analysis at 1-week or 4-, 6- or 8-weeks after the saline or HPβCD treatments. All experimental procedures were approved by the Institutional Animal Care and Use Committee (HER05080Y) at the University at Buffalo in accordance with NIH guidelines.

### Cochlear Preparations

Rats were anesthetized with ketamine (50 mg/kg, i.p.) and xylazine (6 mg/kg, i.p), decapitated and the bullae quickly removed. Openings were carefully made in the cochlear apex, round window, and the oval window and then the cochleae were perfused with 10% formalin in phosphate buffered saline (PBS) and immersed in the fixative overnight.

### Cochleograms

One cochlea from each animal was stained with Ehrlich’s hematoxylin solution as previously described (Johnson et al., 2017). Afterward, the samples were mounted as a flat surface preparation in glycerin on glass slides, coverslipped and examined with a light microscope (Zeiss Standard, 400X). IHCs, OHCs, and pillar cells (PCs) were counted along successive 0.12–0.24 mm intervals from the apex to the base of the cochlea by a second person blind to the experimental conditions. A hair cell or PC was counted as present if both the cuticular plate and cell nucleus were clearly visible and considered missing if either were absent. The percentages of missing IHCs, OHCs and PCs were determined for each animal based on lab norms and a cochleogram constructed showing the percentage of missing OHCs, IHCs, and PCs as a function of percent distance from the apex of the cochlea. Position in the cochlea was related to frequency using a rat tonotopic map (Müller, 1991). Mean percent OHC, IHC, and PC losses were computed over 5% intervals of the cochlea.

### Immunolabeling

In Experiment 1, cochleae from postnatal rats were incubated for 24 h with a primary rabbit anti-SLC26A5 antibody (Millipore Sigma, AV44176, 1:100 dilution) and then incubated for 2 h in a fluorescently labeled (Alexa Fluor 555) goat anti-rabbit secondary antibody (Abcam, ab15008, 1:100 dilution). The specimens were then doubled-labeled with for 1 h with Alexa Fluor 488 conjugated phalloidin (1:200, Invitrogen 474685) to label the hair cell stereocilia and cuticular plate. Specimens were mounted on glass slide, cover slipped, and examined under a confocal microscope with appropriate filters as described previously (Ding et al., 2010).

### Spiral Ganglion Neurons and Auditory Nerve Fibers

To determine if HPβCD resulted in degeneration of SGNs and NFs fibers in the habenula perforata, the temporal bones from rats in Experiment 3 were fixed with 0.25% glutaraldehyde in phosphate-buffered saline for 2 h and then treated with 2% osmium tetroxide for 2 h as described in earlier publications (Ding et al., 1999; Wang et al., 2003). The temporal bones were decalcified for 48 h (Decal, Baxter Scientific Immunoreactivity Products), dehydrated through a graded series of ethanol solutions ending at 100% and then embedded in Epon 812 resin. The Epon-embedded blocks were cut parallel to the axis of the cochlear modiolus at a thickness of 4 μm using an ultramicrotome (Reichert Supernova) equipped with glass knives. Serial sections were collected on glass slides, stained with toluidine blue and examined under a light microscope (Zeiss Axioskop). Specimens were photographed with a digital camera (SPOT Insight, Diagnostic Instruments Inc.) and processed with imaging software (SPOT Software, version 4.6; Adobe Photoshop 5.5). Sections cut tangential to the habenula perforata were used to count the number of fibers passing through each habenula perforata canal. For each experimental condition, the numbers of nerve fibers were counted from 10 habenula perforata canals located in the middle of the basal turn (∼70%-distance-from-apex) of the cochlea of each animal. Measurements were obtained from each animal in the control group (*n* = 6) and each animal in the groups treated with 1,000, 2,000, 3,000, or 4,000 mg/kg HPβCD (*n* = 6/group) (Ding et al., 1999; Fu et al., 2012). Sections cut through the modiolus were used to count the number of SGNs in Rosenthal’s canal in the middle of the basal turn (∼70%-distance-from-apex) of the cochlea as described previously (Ding et al., 1998; McFadden et al., 1999; Fu et al., 2012). For each experimental condition, the number of SGNs in Rosenthal’s canal was counted in each section; data were obtained from five separate sections in each animal (sections separated by 20 μm) and a mean value was computed for each animal. Mean numbers of SGNs per section were computed for each experimental group and the Control group (*n* = 6/group).

### DPOAE and CAP

Our procedures for measuring DPOAEs and CAPs have been described in a recent publication (Liu et al., 2020). The rats in Experiment #2 that were treated with a single 4,000 mg/kg dose of HPβCD at P5, P10, or P15 and the saline Control group were evaluated 8-weeks post-treatment. The animals were anesthetized with ketamine (100 mg/kg, i.p.) and xylazine (20 mg/kg, i.p.), placed on a heating pad to maintain normal body temperature and DPOAEs were measured with an Extended-Bandwidth Acoustic Probe System (ER10X, Etymotic Research, Elk Grove Village, IL, United States) containing two loudspeakers and a low-noise microphone. Custom software was used to generate pairs of primary tones, f1 and f2, and the microphone was used to measure the primary tones and DPOAEs in the ear canal. The higher frequency, f2, of the pair of tones was presented at a frequency 1.2 times greater than the lower frequency, f1 (i.e., f2/f1 = 1.2). The intensity of the lower frequency, L1, was set 10 dB greater than the intensity of the higher frequency, L2. The stimuli were presented for 90 ms at the rate of 5 Hz and repeated 32 times. The acoustic signal in the ear canal was recorded with the low-noise microphone, digitized (RME Babyface Pro, 192 kHz sampling rate, 24 bit A/D converter) and the intensity of f1, f2 and 2f1-f2 calculated using a fast Fourier transform (FFT). DPOAE input/output functions were constructed at f2 frequencies of 4, 8, 16, 24, 30, and 45 kHz by plotting the amplitude of 2f1-f2 at L2 intensities from 15 to 70 dB SPL in 5 dB SPL steps. DPOAEs were measured eight weeks after HPβCD treatment.

Our procedures for recording the CAP are described in a recent publication (Liu et al., 2020). Tone bursts were generated (5 ms duration, 1 ms rise/fall time, cosine2-gated, 2–60 kHz, TDT RX6 Multifunction Processor, 200 kHz sampling rate) and sent to a half-inch condenser microphone/sound source (ACO ½” microphone driven in reverse). The sound source, located within a speculum-like assembly was inserted in the ear canal. The sound source was calibrated with the aid of a ½” microphone (model 2540, Larson Davis), microphone preamplifier (Model 2221, Larson Davis) and A/D converter (RME Babyface Pro) connected to a personal computer and custom sound calibration software.

Rats were anesthetized (ketamine/xylazine cocktail; 100 mg/kg, i.p./30 mg/kg, i.p.), mounted in a custom head-holder, and placed on a temperature controlled heating blanket (Harvard Apparatus). The right cochlea was surgically opened and a recording electrode (Teflon-coated gold wire) was placed on the round window membrane and a silver chloride ground electrode placed in the neck muscle. The electrode output was amplified (1000X, DAM-50 amplifier, WPI), filtered (0.1 Hz–10 kHz), delivered to an A/D converter (100 kHz sampling rate, TDT RX6 Multifunction Processor) and averaged (100X) using custom data acquisition and filtering software (MATLAB 6.1) optimized to exclude the cochlear microphonic potential and isolate the CAP. CAP amplitude was defined as the voltage difference between the first negative peak (N1) and following positive peak (P1). CAP amplitude was plotted as a function of stimulus intensity level to construct CAP input/output functions at 4, 8, 16, 24, 30, and 45 kHz.

### Analysis

GraphPad Prism (ver. 5) and/or Sigma Stat (ver. 3) software were used to plot and/or analyze the results as described below. HPβCD-induced losses of SGNs and ANFs were evaluated using a one-way ANOVA followed by Tukey’s post-hoc comparison.

This research was approved by the University at Buffalo Institutional Animal Care and Use Committee and carried out in accordance with NIH Guidelines.

## Results

### Prestin Is Developmentally Upregulated Along the Cochlear Tonotopic Axis in Rat OHCs

**FIGURE 1:**
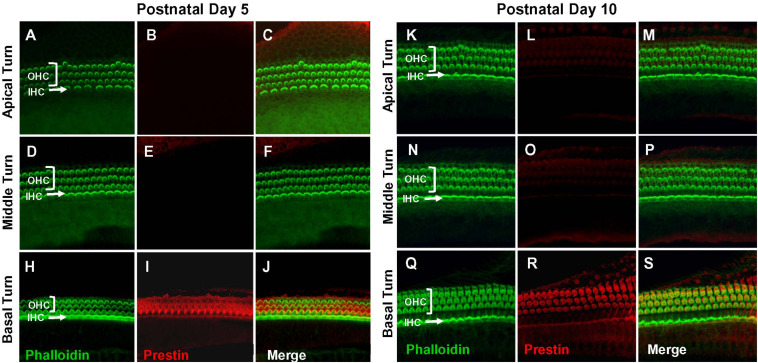
Prestin is only expressed in the basal turn of normal rats on postnatal day 5 (P5) and day 10 (P10). Representative phalloidin-labeled images, prestin-labeled images and merged confocal images of cochlear surface preparations from the apical, middle and basal turn of normal P5 rats **(A–J)** and P10 rats **(K–S)**. **(I,R)** Note strong prestin immunolabeling in basal turn outer hair cells (OHCs), but absence of prestin labeling in inner hair cells (IHCs).

**FIGURE 2:**
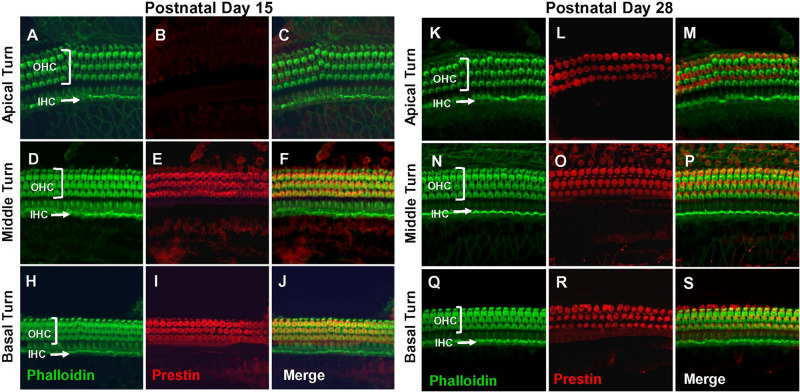
Prestin labeling present in outer hair cells (OHCs) in basal and middle turn at P15 and labeling spreads to OHCs in apical turn at P28. Representative phalloidin-labeled, prestin-labeled and merged confocal images of cochlear surface preparations from the apical, middle and basal turn of normal P15 rats **(A–J)** and P28 rats **(K–S)**. **(I,R)** Note strong prestin immunolabeling in OHCs at P15 and P28, but absence of prestin labeling in inner hair cells IHCs.

Prestin immunolabeling was used to identify the location and relative abundance of prestin in P5, P10, P15, and P28 rats. Strong prestin immunolabeling was only evident in OHCs in the basal turn of the cochlea at P5 (Figures 1A–J) and P10 (Figures 1K–S). However, prestin expression spread to OHCs in the middle turn at P15 (Figures 2A–J) and to OHCs in the apical turn at P28 (Figures 2K–S) consistent with previous reports related to the spatiotemporal developmentally upregulated expression of prestin in OHCs in rats and other rodents (Belyantseva et al., 2000; Jensen-Smith and Hallworth, 2007).

### DPOAE Deficits Greater When HPβCD Administered at P15 Than P5

**FIGURE 3:**
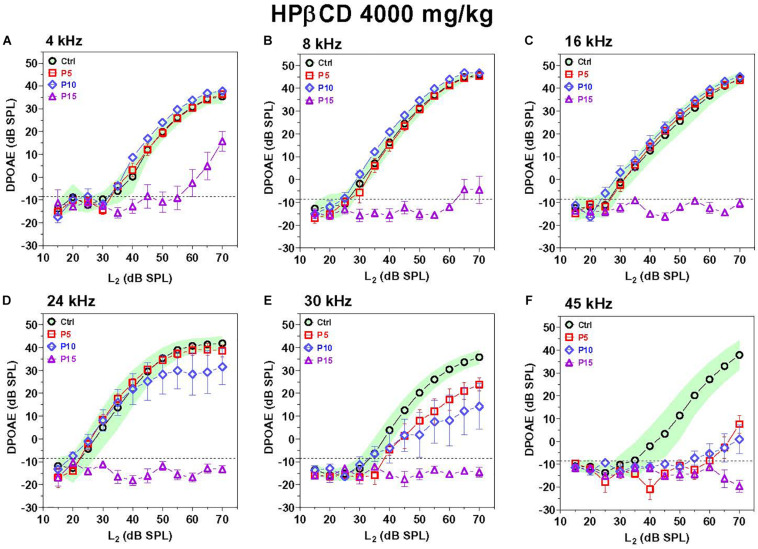
DPOAE deficits are more severe in adulthood if HPβCD is administered to postnatal rats at P15 than P5 or P10. Mean DPOAE input/output functions measured in the adult Control group (*n* = 10, +/-95% confidence interval) compared to mean (+/–SEM, *n* = 6/group) DPOAE input/output functions measured in adults 8-weeks after the rats were treated with 4,000 mg/kg HPβCD at P5, P10 or P15. Data shown for **(A)** 4 kHz, **(B)** 8 kHz, **(C)** 16 kHz, **(D)** 24 kHz, **(E)** 30 kHz and **(F)** 45 kHz.

The exquisite sensitivity and frequency selectivity of the auditory system is dependent on the nonlinear, electromotile properties of OHCs, which contribute to the generation of distortion products that can be detected in the ear canal using DPOAEs (Brownell, 1990; Liberman et al., 2002). Thus, DPOAEs provide a non-invasive method for assessing OHC function. DPOAEs were evaluated 8-weeks after P5, P10, and P15 rats were treated with 4,000 mg/kg of HPβCD or in P5 rats treated with saline. To aid in the interpretation of the results, the mean (*n* = 10) DPOAE input/output functions of the Control group are presented with their 95% confidence intervals and compared to the mean (*n* = 6/group, +/–SEM) input/output functions obtained from the groups treated with HPβCD at P5, P10, or P15 HPβCD (Figure 3). The DPOAEs in the group treated with HPβCD at P15 were largely abolished at frequencies from 8 to 45 kHz and greatly reduced at 4 kHz. In contrast, the groups treated with HPβCD at P5 and P10 had DPOAE input/output functions at 4, 8, and 16 kHz that were completely normal (Figures 3A–C). The P5 DPOAE input/output function at 24 kHz was within normal limits whereas P10 DPOAE amplitudes were slightly below normal at high intensities (Figure 3D). P5 and P10 DPOAE amplitudes at 30 kHz were far below normal; however, the deficits were consistently less for P5 than P10 (Figure 3E). P5 and P10 DPOAE amplitudes were barely detectable except at L2 intensities greater than 60 dB SPL. Thus, HPβCD almost completely eliminated DPOAEs in the P15 group whereas in the P5 and P10 groups it only reduced DPOAE amplitudes at the high, but not the low frequencies.

### CAP Deficits Greater When HPβCD Administered at P15 Than P5

**FIGURE 4:**
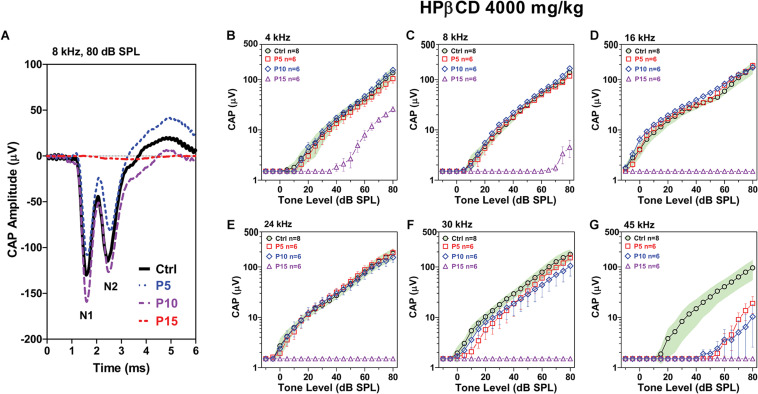
Adult CAP deficits are more severe when HPβCD is administered to postnatal rats at P15 than P5 or P10. **(A)** Representative CAP waveforms elicited by 8 kHz tone bursts presented at 80 dB SPL. Note large reduction in CAP amplitude in adult rats when HBβCD was administered at P15 and nearly normal response when the drug was administered at P5 or P10. Mean (*n* = 8, +/-95% confidence interval) CAP input/output functions at **(B)** 4 kHz, **(C)** 8 kHz, **(D)** 16 kHz, **(E)** 24 kHz, **(F)** 30 kHz and **(G)** 45 kHz in Control rats compared to mean (+/–SEM, *n* = 6/group) input/output functions obtained 8-weeks after the rats were treated with 4000 mg/kg HPβCD at P5, P10 or P15. CAP amplitudes in the P5 and P10 group were much larger than those in the P15 group. At 45 kHz, CAP amplitudes in the P5 and P10 group were much smaller than in the Control group.

Type I auditory nerve fibers make one to one synaptic contact with IHCs. Tone burst-evoked activation of IHCs results in the sudden release of neurotransmitter from the base of the IHC onto afferent terminals resulting in synchronous neural discharges that generate the CAP (Bourien et al., 2014). The CAP, which reflects the neural output of the cochlea relayed from the IHCs, was measured 8-weeks after the P5, P10, and P15 rats had been treated with 4,000 mg/kg of HPβCD and compared to measurements from rats in the saline Control. The CAP waveforms elicited by 80 dB SPL tone bursts presented at 8 kHz are shown in Figure 4A; results are presented for a representative rat in the saline Control group and rats treated with HPβCD at P5, P10, or P15. The CAP measurement were obtained 8-weeks post-treatment. Large N1 and N2 responses were present in the Control, P5 and P10 rats, whereas N1 and N2 response were absent in the P15 rat. To visualize the age-dependent effects of the HPβCD treatment, the mean (*n* = 8) CAP input/output functions of the Control group are presented with their 95% confidence intervals and the results compared to the mean (n = 6/group, +/–SEM) CAP input/output functions measured 8-weeks after the P5, P10, and P15 groups had been treated with 4,000 mg/kg HPβCD (Figures 4B–G). CAP responses in the P15 group were largely abolished from 8 to 45 kHz; the CAP was present at 4 kHz, but the amplitude was greatly reduced (Figure 4B). In contrast, CAP responses in the P5 and P10 group were similar to the Control group from 4 to 24 kHz (Figures 4B–E), slightly reduced at 30 kHz (Figure 4F) and greatly reduced at 45 kHz compared to the Control group (Figure 4G). The deficits at 45 kHz were slightly greater at P10 than at P5 (Figure 4G). HPβCD was thus much more ototoxic when administered at P15 than P5 or P10 and the ototoxic effects were more severe at the high frequencies.

### Cochlear Lesions Greater When HPβCD Administered at P28 Than P10 or P15

**FIGURE 5:**
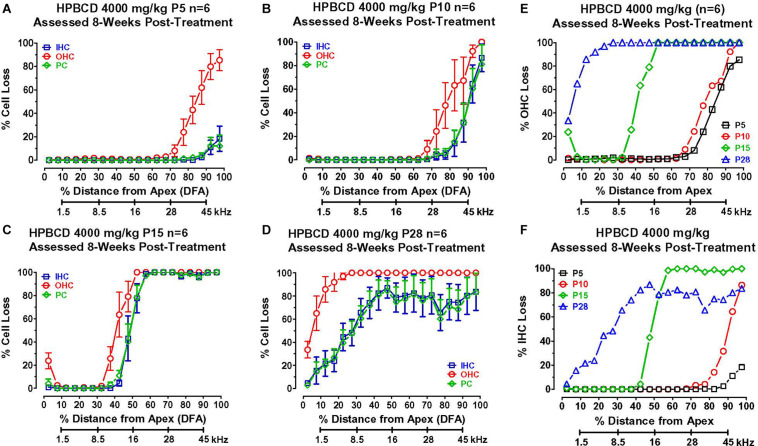
Mean (+/–SEM, *n* = 6) cochleograms showing percent missing outer hair cells (OHCs), inner hair cells (IHCs), and pillar cells (PCs) plotted as function of percent distance from the apex (DFA). Frequency-place map of rat cochlea below abscissa (Müller, 1991). Data obtained 8-weeks after **(A)** P5, **(B)** P10, **(C)** P15, and **(D)** P28 rats were treated with 4,000 mg/kg of HPβCD. Large OHC lesion present 70–100% DFA at P5 and P10 **(A,B)**. OHC lesion spreads toward the apex from P15 **(C)** to P28 **(D)**. Minimal IHC and PC losses in base of cochlea at P5 **(A)**. IHC and PC losses increase dramatically and spread from the base toward the apex of the cochlea from P10 to P28. Comparison of mean (*n* = 6/group) **(E)** OHC loss and **(F)** IHC loss when HPβCD administered at P5, P10, P15 or P28. Note rapid expansion of OHC and IHC losses from P5 to P28.

To determine if the cochlear histopathologies from HPβCD treatment were age-dependent, mean cochleograms (*n* = 6/group, +/–SEM) were prepared 8-weeks after the P5, P10, P15, and P28 rats had been treated with 4,000 mg/kg of HPβCD. The smallest cochlear pathologies occurred in the group treated with HPβCD at P5 (Figure 5A). The OHC losses in this group decreased from 100% at the base to less than 10% approximately 70% distance from the apex (DFA). Small (<25%) IHC and PC losses were present 90–100% DFA. The OHC losses in the P10 group were similar to those seen in the P5 group (Figure 5B), but the IHC and PC losses in the P10 group, which were more severe than at P5, approached 90% at the most basal location. The cochlear lesions were much more extensive and severe in the P15 group (Figure 5C). All of the OHCs were missing over the basal half of the cochlea followed by a decrease in OHC loss to less than 10% loss ∼30% DFA. All of the IHCs and PCs were missing over the basal two-thirds of the cochlea; the loss decreased to less than 10% approximately 45% DFA. There was a further apical expansion of the lesion in the group treated with HPβCD at P28 (Figure 5D). The OHC lesion decreased from 100% over the basal two-thirds of the cochlea to ∼30% at the extreme apex. Unlike the P15 group, the IHC and PC lesions in the P28 group hovered around 80% over the basal two-thirds of the cochlea followed by a gradual decline to less than 10% IHC and PC loss in the extreme apex. Thus, the OHC lesion was confined to the base of the cochlea when HPβCD was administered at P5 or P10 and the OHC lesion was only slightly greater at P10 than P5. The OHC lesion rapidly expanded toward the apex when the drug was administered from P15 to P28 (Figure 5E), a pattern consistent with the developmental upregulation of prestin expression in rat OHCs (Belyantseva et al., 2000). HPβCD also caused extensive damage to IHCs and PCs, but the damage was restricted to cochlear locations where the drug also caused at least 30% or more OHC loss. IHC and PC damage was never observed at cochlear locations where nearly all the OHCs were present. The IHC lesion also rapidly expanded toward the apex when HPβCD was administered at P15 or P28 (Figure 5F).

### Delayed Onset Between OHC Loss and IHC and PC Degeneration

**FIGURE 6:**
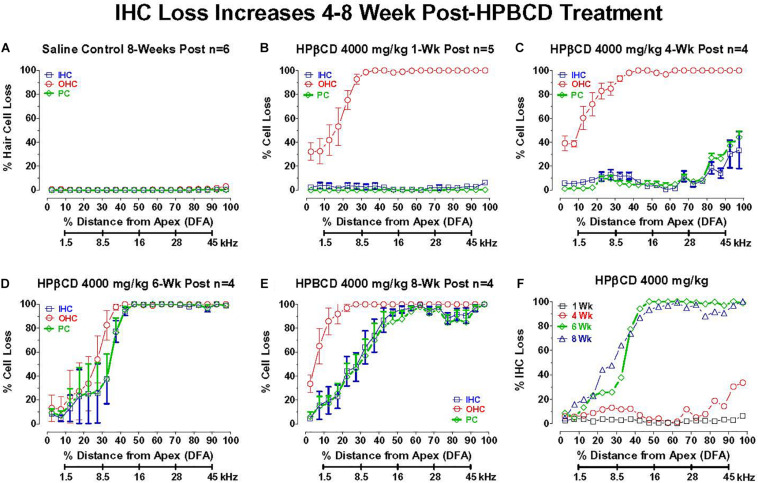
Inner hair cell (IHC) and pillar cell (PC) losses dramatically increase in adult rats 4–8 week after 4,000 mg/kg HPβCD treatment. Mean (+/–SEM) cochleograms showing percent missing OHCs, IHCs and pillar cells (PC) plotted as function of percent distance from the apex (DFA) in adult rats. Rat cochlear frequency-place map shown below abscissa (Müller, 1991). **(A)** Mean (*n* = 6) data from saline Control group 8-weeks post-treatment (*n* = 6). Mean cochleograms obtained from adult rats treated with 4,000 mg/kg HPβCD and evaluated **(B)** 1-week post-treatment (*n* = 5), **(C)** 4-weeks post-treatment (*n* = 4) **(D)** 6-weeks post-treatment (*n* = 4), and **(E)** 8-weeks post-treatment (*n* = 4). Massive OHC loss 1-week after 4,000 mg/kg treatment, but no IHC and PC loss **(B)**, but massive IHC and PC loss occurs 6- to 8-weeks post-treatment **(D,E)**. **(F)** Mean cochleogram showing percent IHC loss as a function of percent distance from the apex (DFA) of the cochlea; cochlear frequency-place map shown on the abscissa. Note large increase in IHC loss between 4- and 6-weeks post-treatment.

**FIGURE 7:**
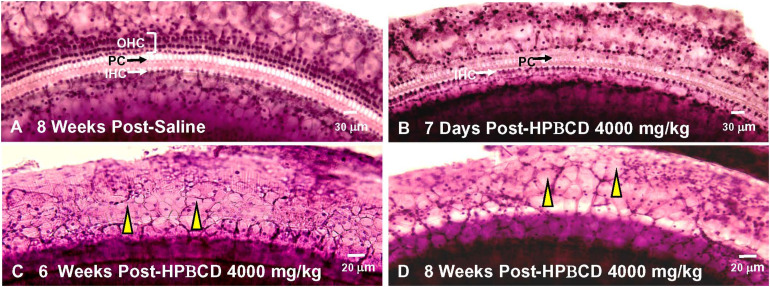
Massive inner hair cell (IHC) and pillar cells(PC) loss occurs 6–8 weeks after treatment with 4,000 mg/kg HPβCD. Representative photomicrographs of hematoxylin and eosin stained cochlear surface preparation from middle of the basal turn (60–80% distance from apex) of the cochlea. Representative photomicrograph shown for **(A)** 8-weeks after saline treatment and **(B)** 1-week, **(C)** 6-weeks, and **(D)** 8-weeks after treatment with 4,000 mg/kg of HPβCD. **(A)** Three orderly rows of outer hair cells (OHC), single row of inner hair cells (IHC) and pillar cells present in saline controls. **(B)** IHCs and PCs present, but most OHCs missing 1-week after treatment with 4,000 mg/kg HPβCD. **(C,D)** Massive OHC, IHC and PC loss results in flattened sensory epithelium that is replaced by many large, cuboidal cells (yellow arrowhead).

2-Hyroxypropyl-beta-cyclodextrin was originally believed to only damage prestin-bearing OHCs (Crumling et al., 2012; Cronin et al., 2015; Takahashi et al., 2016). However, more recent work indicates that HPβCD also leads to the delayed destruction of IHCs and PCs (Liu et al., 2020). To determine when the IHCs and PCs degenerated, we treated adult rats with 4,000 mg/kg of HPβCD or saline and evaluated their cochleae from 1- to 8-weeks post-treatment. The mean cochleogram (*n* = 6) from the saline Control group showed no loss of OHCs, IHCs, or PCs 8-weeks post-treatment (Figure 6A) whereas massive OHC loss was evident over most of the cochlea 1-week after HPβCD treatment (Figure 6B). The magnitude and extent of the OHC lesion was roughly the same at 4-, 6-, and 8-weeks post-treatment (Figures 6C–E). There was little evidence of IHC or PC loss 1-week after HPβCD treatment. After 4-weeks, a mild IHC/PC lesion emerged near the base of the cochlea, but at 6-weeks post-treatment the IHC/PC lesion underwent a massive expansion to match the large OHC pathology (Figure 6D). A large IHC/PC lesion that followed the general contour of the OHC pathology was also present 8-weeks post-treatment. To illustrate the delayed, but rapid expansion of IHC lesion between 4- and 6-weeks, Figure 6F compares the mean IHC lesion at 1-, 4-, 6-, and 8-week(s) post-treatment. The massive IHC/PC lesions present at 6- and 8-weeks post-treatment were associated with a flattened sensory epithelium composed of large cuboidal cells (Figures 7C,D) that had replaced the highly stereotyped network of sensory cells and support cells that comprise the partially damaged (Figure 7B) or normal organ of Corti (Figure 7A).

### Delayed Nerve Fiber and Spiral Ganglion Degeneration

**FIGURE 8:**
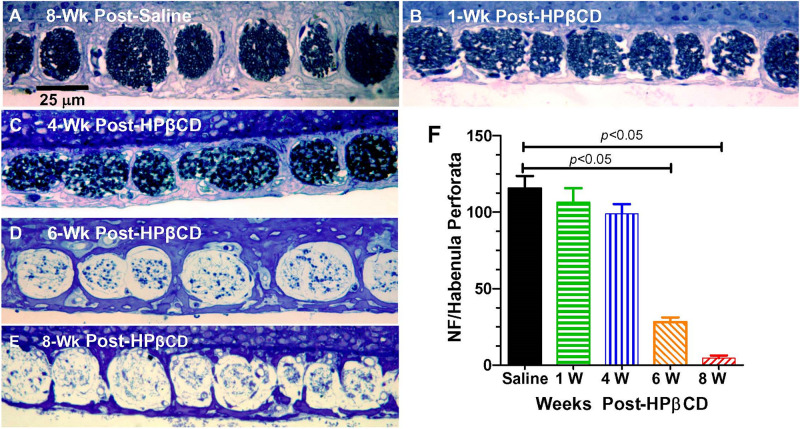
Massive loss of nerve fibers occurs in habenula perforata 6- to 8-weeks following 4,000 mg/kg treatment with HPβCD. Representative photomicrograph of methylene blue-stained 4 micron sections taken tangent to the habenula perforata in the middle of the basal turn from an adult rat in the **(A)** saline Control group 8-weeks post-treatment and from adult rats treated with 4,000 mg/kg HPβCD and evaluated **(B)** 1-week, **(C)** 4-weeks, **(D–E)** 6- and 8-weeks post-treatment. Nerve fibers densely packed in habenula perforata of **(A)** saline control; nerve fiber packing density slightly reduced at 1- and 4-week post-treatment followed by a massive reduction in nerve fiber density 6- and 8-weeks post-treatment **(C,D)**. **(F)** Mean numbers (+SEM, *n* = rats/group) of nerve fibers per habenula perforata in saline Control group and groups treated with 4,000 mg/kg HPβCD and evaluated 1-, 4-, 6- or 8-weeks post-treatment.

Massive loss of hair cells and support cells often leads to the degeneration of nerve fibers that innervate the cochlea (McFadden et al., 2004). To determine if and when the nerve fibers were degenerating after administering 4,000 mg/kg of HPβCD, we counted the number of nerve fibers in the habenula perforata in the basal turn of the cochlea 1-, 4-, 6-, and 8-week(s) after treatment with 4,000 mg/kg of HPβCD. The habenula perforatae were densely packed with nerve fibers in the Control cochlea (Figure 8A) and there was only a slight hint of a reduction in fiber density at 1- and 4-weeks post-treatment (Figures 8B,C); however, there was a massive reduction in fiber density in the habenula perforatae at 6- and 8-weeks post-treatment (Figures 8D,E). The mean (*n* = 6/group, +SEM) number of nerve fibers per habenula perforata was approximately 116 (*n* = 6 rats) in the saline Control group (Figure 8F). The mean (*n* = 6 rats/group) numbers of nerve fibers decreased to 106, 99, 28, and 5, respectively when rats were evaluated at 1-week or 4-, 6-, or 8-week(s) after treatment with 4,000 mg/kg HPβCD. The numbers of nerve fibers in the 6- and 8-weeks groups were significantly less than those in the Control group [one-way analysis of variance (*F* (4, 25) = 62.47, *p* < 0.0001, Tukey’s multiple comparison, *p* < 0.05].

**FIGURE 9:**
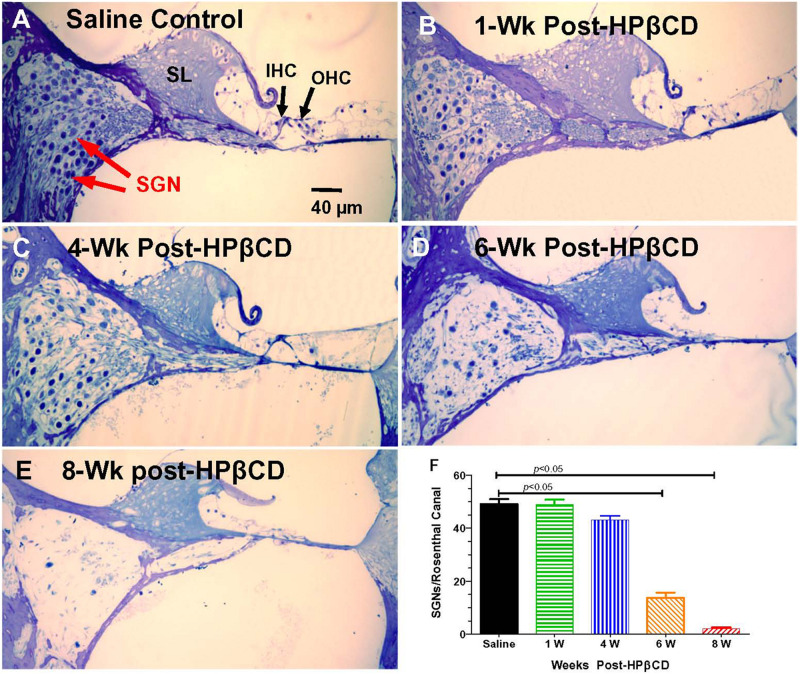
Massive loss of spiral ganglion neurons (SGN) in Rosenthal’s canal 6- to 8-weeks following 4,000 mg/kg treatment with HPβCD. Thin sections (4 μm) through the modiolus in the middle of the basal turn of the cochlea. **(A)** Saline control showing inner hair cell (IHC), outer hair cells (OHC), and spiral limbus (SL). Rosenthal’s canal filled with SGNs. **(B–E)** Sections from adult rats treated with 4,000 mg/kg of HPβCD and evaluated 1-, 4-, 6- and 8-weeks post-treatment: Numbers of SGNs in Rosenthal’s canal **(B)** normal at 1-week post-treatment, **(C)** slightly reduced at 4-weeks post-treatment and greatly reduced at **(D)** 6-weeks and **(E)** 8-weeks post-treatment. **(F)** Mean (*n* = 6, +SEM) numbers of SGNs in Rosenthal’s canal in the saline Control group versus the groups treated with 4,000 mg/kg HPβCD and evaluated 1-, 4-, 6- and 8-weeks post-treatment. Numbers of SGNs in HPβCD-treated groups evaluated at 6- and 8-weeks post-treatment were significantly less than in the saline Control group.

The nerve fiber loss in the habenula perforatae was accompanied by a corresponding loss of SGNs. Rosenthal’s canal in the middle of the basal turn of the cochlea was packed with SGNs in the saline Control group together with IHCs and OHCs in the organ of Corti adjacent to the spiral limbus (SL) (Figure 9A). The OHCs were missing 1-week after treatment with 4,000 mg/kg of HPβCD, but numerous SGNs were still present in Rosenthal’s canal (Figure 9B). The modest reduction of SGNs in Rosenthal’s canal at 4-weeks post-treatment was followed by a massive loss at 6-weeks post-treatment (Figure 9D) and a nearly complete loss at 8-weeks post-treatment (Figure 9E). Despite the massive loss of hair cells, SGNs and collapse of the organ of Corti, the SL appeared relatively normal (Figure 9D). The loss of SGNs was quantified in the different groups in the middle of the basal turn. The mean (*n* = 6, +SEM) number of SGNs per Rosenthal’s canal was approximately 49 in both the saline Control group and the group evaluated 1-week after treatment with 4,000 mg/kg HPβCD (Figure 9F). The mean (*n* = 6 rats/group, +SEM) number decreased slightly to 43 at 4-weeks post-treatment followed by a dramatic reduction to 14 at 6-weeks and nearly a complete loss of SGNs at 8-weeks post-treatment. The numbers of SGNs in the 6- and 8-weeks groups were significantly less than in the Control group (one-way analysis of variance (*F* (4, 25) = 179.9, *p* < 0.0001, Tukey’s multiple comparison, *p* < 0.05).

## Discussion

### Prestin Upregulation Determines Spread of High-Frequency Auditory Impairment

2-Hyroxypropyl-beta-cyclodextrin-induced hearing loss is associated with the rapid destruction of OHCs (Crumling et al., 2012, 2017; Cronin et al., 2015; Ding et al., 2020, 2021). This rapid destruction of OHCs can be tracked non-invasively by serially measuring DPOAEs. In adult rats, the 4,000 mg/kg dose of HPβCD completely eliminated the DPOAE 2-days post-treatment (Liu et al., 2020). Although several mechanisms have been suggested (Crumling et al., 2017), selective cyclodextrin ototoxicity is believed to be correlated with prestin expression in OHCs because knockout mice missing this protein are more resistant to cyclodextrin damage than wild type mice (Takahashi et al., 2016) or mutant mice with a non-electromotile form of prestin (Zhou et al., 2018).

In order to investigate the developmental upregulation of prestin in OHCs from base to apex, we employed a high 4,000 mg/kg dose of HPβCD that would destroy OHCs over the entire length of the rat cochlea (Liu et al., 2020). The functional deficits observed in our postnatal rats treated with HPβCD were closely correlated with the temporal and spatial upregulation of prestin in OHCs indicating that prestin, or some other unknown factor associated with its expression, is critically important for initiating cyclodextrin-induced OHC loss. If HPβCD was administered to rats at P5 or P10, when prestin was absent from OHCs in the apical and middle turn, then low- and mid-frequency DPOAEs and CAPs measured 8-weeks post-treatment were normal, i.e., the absence of prestin in OHCs when HPβCD was administered prevented cyclodextrin ototoxicity at the low- and mid-frequencies. Prestin was already present in basal turn OHCs at P5 and P10 and when rats were treated with HPβCD at these ages, high-frequency DPOAEs and CAPs measured 8-weeks post-treatment were greatly depressed. When HPβCD was administered to rats at P15, when prestin was also expressed in middle-turn OHCs, then mid-frequency DPOAEs and CAPs measured 8-weeks post-treatment were also depressed. Thus, the developmental spatiotemporal upregulation of prestin in OHCs determined when the OHC loss and cochlear hearing loss spread from high to low frequencies.

The postnatal time at which HPβCD was administered together with the spatiotemporal expression of prestin determined the location and breadth of the HPβCD-induced OHC lesion (Figure 5F). The OHC lesion was confined to the high-frequency, basal third of the cochlea when HPβCD was administered at P5 or P10, consistent with the presence of prestin in the base of the cochlea. However, the OHC lesion expanded to cover the basal two-thirds of the cochlea when cyclodextrin was administered at P15, at which time prestin expression had expanded from the base to the middle turn of the cochlea. Importantly, prestin was expressed in the apical turn, as well as middle and basal turns, at P28. Only then did the OHC lesions appear in the most apical region of the cochlea. Thus, the expansion of the OHC lesion toward the apex of the cochlea was closely correlated with the developmental base to apex upregulation of prestin, which finally appeared in the cochlear apex at P28. Our apical turn results might account for the lack of OHC loss in the apex of the cochlea of WT mice treated with 8000 mg/kg of HPβCD on P21 (Takahashi et al., 2016). The absence of OHC damage in the apex of the WT mouse cochlea is most likely due to the fact that prestin was expressed at such a low level on P21 that HPβCD was unable to damage OHCs in the most apical portion of the cochlea.

### Mechanisms of HPβCD OHC Damage

Several different mechanisms could contribute to the cyclodextrin-induced OHC death (Crumling et al., 2012, 2017), but interactions between cholesterol and prestin have garnered the most attention. Prestin is expressed in the lateral wall of mature OHCs and has also been reported in the cytoplasm of vestibular hair cells although its cytoplasmic expression has not been independently verified (Belyantseva et al., 2000; Adler et al., 2003). Systemic administration of cyclodextrin, however, does not damage adult vestibular hair cells (Ding et al., 2020, 2021), nor does it damage postnatal OHCs lacking prestin. Prestin is heavily expressed along the OHC lateral wall, adjacent to the actin-spectrin cortical lattice and subsurface cisternae (Jensen-Smith and Hallworth, 2007; Legendre et al., 2008). Prestin appears to interact with cholesterol (Takahashi et al., 2016) and experimental manipulations of cholesterol affect OHC stiffness, electromotility, the lateral mobility of prestin in the membrane and prestin function (Nguyen and Brownell, 1998; Rajagopalan et al., 2007; Kamar et al., 2012). Imaging studies have revealed high levels of cholesterol staining at the apical and basal poles of normal OHCs. In contrast, low-levels of cholesterol are present along the lateral wall of OHCs in WT mice whereas cholesterol labeling is more abundant in the lateral wall of prestin knockout mice (Nguyen and Brownell, 1998; Rajagopalan et al., 2007; Brownell et al., 2011; Takahashi et al., 2016). Treatment of isolated OHCs with cyclodextrin often leads to rapid cell death associated with rupture of the cell’s membrane. In contrast, OHCs in prestin knockout mice are largely resistant to cyclodextrin (Rajagopalan et al., 2007; Takahashi et al., 2016). In cochlear organotypic cultures, HPβCD-induced OHC death was initiated by the extrinsic caspase-8 apoptotic pathway that is triggered by ligands that bind to death receptors on the cell’s membrane (Salvesen, 2002; Cheng et al., 2005; Ding et al., 2020). Because of the low-abundance of cholesterol in the lateral wall of normal OHCs, further cyclodextrin-induced depletion of cholesterol from this region likely leads to the breakdown of the plasma membrane in the lateral wall of OHCs leading to rapid onset of caspase-8 mediated cell death (Garofalo et al., 2003; Schonfelder et al., 2006). Caspase-8 inhibitors could be used to test this hypothesis (Wang et al., 2004; Bozzo et al., 2006).

### Time Course of IHC, PC, and SGN Degeneration

Much of the previous literature on HPβCD ototoxicity in mice has focused on its early, selective destruction of OHCs with little evidence of damage to IHCs, ANFs, or SGNs (Crumling et al., 2012, 2017; Takahashi et al., 2016; Zhou et al., 2018). However, recent studies from our lab have revealed that HPβCD unexpectedly causes massive, delayed degeneration of rat IHCs, PCs, ANFs, SGNs, and much of the organ of Corti (Ding et al., 2020; Liu et al., 2020). The abrupt onset of this massive secondary loss of IHCs, ANFs, and SGNs differs from the progressive loss of SGNs seen after most other ototoxic drug insults or traumatic noise exposures (Koitchev et al., 1982; Bichler et al., 1983; Yagi et al., 2000; Lin et al., 2011). The abrupt onset of this second wave of degeneration may have been missed in previous mouse studies because the bulk of the damage occurred well beyond the time period when degeneration of OHCs and IHCs typically occurs. From our previous studies, it was unclear exactly when the IHC, ANF, and SGN damage occurred. Our results indicate that this secondary degenerative process occurs suddenly, starting in the base of the cochlea 4-weeks post-treatment and expanding to virtually all of the cochlea by 8-weeks post-treatment (Figures 5, 8, 9). The sudden increase in these IHC, ANF and SGN pathologies occurred between 4- and 6-weeks post-treatment. We are unaware of any ototoxicity studies showing an abrupt, massive upsurge of IHC, ANF and SGN damage more than a month after the initial damage to OHCs occurs. In some cases, nearly 100% of the IHCs and PCs were missing in regions with complete OHC loss (Figure 6), but in other cases only ∼80% had degenerated (Figure 5). Additional studies are needed to determine if all the IHCs, support cells and SGNs eventually degenerate with longer survival times or if the degenerative processes are essentially complete by 8-weeks post-treatment. Although high doses of HPβCD are highly ototoxic, studies have shown that very little of the drug actually crosses the blood-brain barrier when it is administered systemically (Ramirez et al., 2011; Pontikis et al., 2013; Vecsernyes et al., 2014). Therefore, the levels of HPβCD in the cochlea would be expected to be low unless the blood-labyrinth barrier was uniquely permeable to HPβCD or if the drug disrupted the blood-labyrinth barrier. Systemically administered HPβCD is rapidly cleared from the body within a few hours; however, its effects on cholesterol synthesis may be long lasting (Aqul et al., 2011) raising the possibility of more prolonged degenerative effects.

2-Hyroxypropyl-beta-cyclodextrin-induced OHC degeneration occurs rapidly within hours after intracerebral administration and within 1 day after subcutaneous dosing (Crumling et al., 2017). The acute damage to OHCs has been repeatedly observed (Figure 6; Crumling et al., 2012; Cronin et al., 2015; Takahashi et al., 2016; Ding et al., 2020). In contrast, the onset of IHC, PC, ANF and SGN degeneration did not begin until 4-weeks post-treatment, but during the following two weeks there was a large increase in the magnitude and apical expansion of the lesion (Figures 6F, 8F, 9F; Liu et al., 2020; Ding et al., 2021). The PC losses were highly correlated with the IHC lesions suggesting that the survival of these two structures depended on some common factor such as loss of neurotrophic support (Mueller et al., 2002; Pickles and Chir, 2002; Fritzsch et al., 2004). Reintroducing one or more neurotrophic factors into the cochlea at specific times following HPβCD treatment could aid in identification of specific neurotrophic factors that enhance the survival of IHCs, PCs, ANFs or SGNs (Atkinson et al., 2012; Chen et al., 2018; Leake et al., 2019; Ma et al., 2019). Cholesterol depletion is known to disrupt intercellular communication and tight junctions and lead to anoikis-like cell death (Lambert et al., 2007; Park et al., 2009; Zhang et al., 2018), disruption of the reticular lamina and/or dedifferentiation of the organ of Corti (Favre and Sans, 1991; Kiernan et al., 2005; Oesterle and Campbell, 2009; Taylor R. R. et al., 2012). In order to account for the delayed degeneration of these structures, HPβCD would need to induce long-lasting changes in sterol homeostasis.

The early, massive loss of OHCs could lead to chronic cochlear neuroinflammation associated with release of toxic substances from dying cells or the subsequent invasion and/or upregulation of phagocytic cells (macrophages/microglia) (Wood and Zuo, 2017). Chronic macrophage/microglia activation begins around 4-weeks after intense noise exposure (Baizer et al., 2015). The proinflammatory molecules which these immune cells release (Dheen et al., 2007) could contribute to the delayed HPβCD-induced IHC, PC, ANF, and SGN degeneration. This hypothesis could be tested by administering therapeutic compounds that suppress neuroinflammation (Lee et al., 2016; Tillinger et al., 2018). Finally, if the secondary wave of IHC, ANF, and SGN degeneration is caused by an inflammatory storm initiated by the massive degeneration of OHCs, then HPβCD-induced damage to these structures would be expected to be minimal in animals in which the OHCs have been already be eliminated by genetic or other manipulations. However, if this secondary wave of degeneration is due to long term disruption of cholesterol homeostasis, then HPβCD would be expected to cause delayed damage (second wave) to IHCs, ANFs and SGNs in animals with missing OHCs.

### Synthesis

**FIGURE 10:**
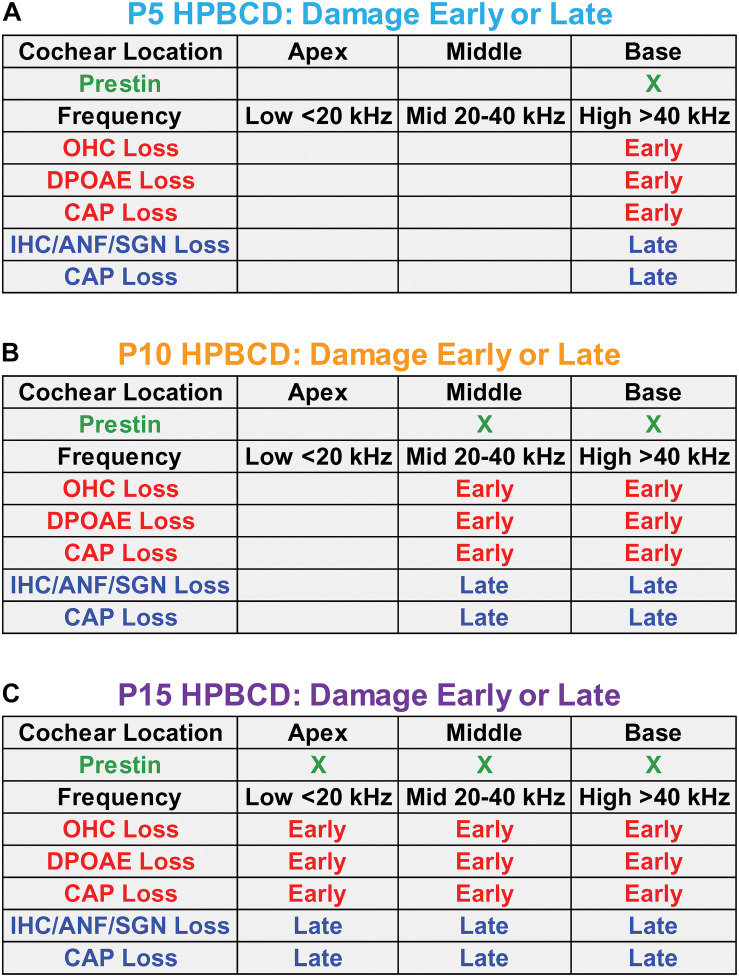
Schematics in panels **(A–C)** showing the approximate location of prestin in outer hair cells (OHCs) in the base, middle, and apex of the cochlea on postnatal day 5, 10, and 15 (P5, P10, and P15) when HPβCD was administered. Each panel shows the approximate cochlear location and frequency of the loss and/or damage to OHCs, distortion product otoacoustic emissions (DPOAEs), the compound action potential (CAP), inner hair cells (IHCs), auditory nerve fibers (ANFs), and spiral ganglion neurons (SGNs) approximately 1-week (Early phase) and approximately 8-weeks (Late phase) after HPβCD treatment. The least damage/loss occurs when HPβCD is administered at P5 when prestin is mainly expressed in the base of the cochlea. The greatest damage/loss occurs when HPβCD is administered at P15 when prestin is expressed over most of the cochlea. CAP loss is observed in the early phase because the OHC and DPOAE losses affect the input to the IHCs. CAP losses are exacerbated during the late phase because of the additional loss of IHCs, ANFs and SGNs.

Figure 10 is a schematic that attempts to synthesize and integrate the general findings of the current study as well as the results from earlier reports (Ding et al., 2020, 2021; Liu et al., 2020). At P5, prestin is most heavily expressed in the base of the cochlea (Figure 10A). When HPβCD is administered at P5, it initially (early phase 1-week post-treatment) destroys only the OHCs in the base of the cochlea eliminating or greatly reducing high frequency DPOAEs. Because of OHC amplification, the input to the IHCs is reduced during the early phase of damage thereby increasing the threshold and reducing the CAP amplitude that is initiated by IHC neurotransmitter release (Dallos and Harris, 1978). Between 4- and 8-weeks after HPβCD treatment, the IHCs, ANFs, and SGNs abruptly die off during the late phase of degeneration; this further exacerbates the CAP loss. If HPβCD is administered at P10 when prestin expression in OHCs expands further toward the middle of the cochlea (Figure 10B), the OHC, DPOAE, and CAP losses expand further toward the apex and to lower frequencies during the early phase of damage (1-week post-treatment). During the late phase, the IHCs, ANFs, SGNs suddenly die off and CAP losses are exacerbated. Now the losses include both high- and mid-frequencies. If HPβCD is administered at P15 or later when prestin in OHCs is expressed throughout the cochlea (Figure 10C), the OHC, DPOAE, and CAP losses expand toward the apex and lower frequencies during the early phase of damage. During the late phase, the IHCs, ANFs, and SGNs also die off and CAP losses are further exacerbated. Thus, the greatest and most widespread damage from HPβCD occurs after prestin expression has reached adult levels.

### Clinical Implications

2-Hyroxypropyl-beta-cyclodextrin ameliorates many of the clinical disorders associated with NPC1 and slows the progression of the disease, but a major side effect is rapid onset sensorineural hearing loss that spreads from high to low frequencies (Ward et al., 2010; Crumling et al., 2012; King et al., 2014; Cronin et al., 2015; Maarup et al., 2015). The initial reports in animals suggested that the HPβCD-induced hearing loss was caused by early damage to the OHCs consistent with the results presented here, but recent results indicate that there is massive, delayed damage to IHCs, ANFs and SGNs that occurs between 4 and 8-weeks post-treatment (Figures 6, 8, 9; Liu et al., 2020; Ding et al., 2021). This second, delayed wave of degeneration implies that HPβCD ototoxicity is worse than previously believed. The clinical consequences of IHC and SGN degeneration is that it greatly reduces the benefits of hearing aids and cochlear implants for patients receiving HPβCD treatments. Given that the delayed degeneration of IHCs, ANFs, and SGNs occurs over a well-defined 4- to 8-week time window following HBβCD treatment, researchers could use this animal model to test out various therapeutic interventions to prevent the secondary degeneration of the remaining IHC, ANFs and SGNs using antioxidants, neurotrophins or a cocktail of the two (Rybak and Whitworth, 2005; Atkinson et al., 2012; Leake et al., 2019; Chen et al., 2020). The optimal time for delivering these therapies would seem to be from approximately 7-days after HPβCD treatment to 8-weeks post-treatment and possibly longer.

## Data Availability Statement

The raw data supporting the conclusions of this article will be made available by the authors, without undue reservation.

## Ethics Statement

The animal study was reviewed and approved by University at Buffalo Institutional Animal Care and Use Committee and carried out in accordance with NIH Guidelines.

## Author Contributions

DD: conceptualization, investigation, methodology, data curation, formal analysis, visualization, writing, and revision. HJ: investigation, methodology, and data curation. SM: conceptualization, methodology, and revision. XL: methodology and software development. LL: investigation, formal analysis, and visualization. G-DC: conceptualization, investigation, methodology, data curation, formal analysis, visualization, and revision. RS: conceptualization, formal analysis, visualization, writing, revision, project administration, and funding acquisition. All authors contributed to the article and approved the submitted version.

## Conflict of Interest

The authors declare that the research was conducted in the absence of any commercial or financial relationships that could be construed as a potential conflict of interest.

## Abbreviations

ANF: auditory nerve fibers
HP β CD: 2-hydroxylpropyl-beta-cyclodextrin
CAP: compound action potential
DPOAE: distortion product otoacoustic emission
IHC: inner hair cells
NF: nerve fiber
NPC1: Niemann-Pick Type C1
OHC: outer hair cells
PC: pillar cells
SGN: spiral ganglion neurons
SL: spiral limbus
WT: wild type.

## References

[B1] Adler H. J. Belyantseva I. A. Merritt R. C. Frolenkov G. I. Jr. Dougherty G. W. Kachar B. (2003). Expression of prestin, a membrane motor protein, in the mammalian auditory and vestibular periphery. *Hear. Res.* 184 27–40. 10.1016/s0378-5955(03)00192-8 14553901

[B2] Aqul A. Liu B. Ramirez C. M. Pieper A. A. Estill S. J. Burns D. K. (2011). Unesterified cholesterol accumulation in late endosomes/lysosomes causes neurodegeneration and is prevented by driving cholesterol export from this compartment. *J. Neurosci.* 31 9404–9413. 10.1523/jneurosci.1317-11.2011 21697390 PMC3134878

[B3] Atkinson P. J. Wise A. K. Flynn B. O. Nayagam B. A. Hume C. R. O’Leary S. J. (2012). Neurotrophin gene therapy for sustained neural preservation after deafness. *PLoS One* 7:e52338. 10.1371/journal.pone.0052338 23284995 PMC3524079

[B4] Baizer J. S. Wong K. M. Manohar S. Hayes S. H. Ding D. Dingman R. (2015). Effects of acoustic trauma on the auditory system of the rat: the role of microglia. *Neuroscience* 303 299–311. 10.1016/j.neuroscience.2015.07.004 26162240 PMC4532607

[B5] Belyantseva I. A. Adler H. J. Curi R. Frolenkov G. I. Kachar B. (2000). Expression and localization of prestin and the sugar transporter GLUT-5 during development of electromotility in cochlear outer hair cells. *J. Neurosci.* 20:RC116. 10.1523/JNEUROSCI.20-24-j0002.2000 PMC6773019 11125015

[B6] Bessell E. Fuller N. R. Markovic T. P. Burk J. Picone T. Hendy C. (2019). Effects of alpha-cyclodextrin on cholesterol control and compound K on glycaemic control in people with pre-diabetes: protocol for a phase III randomized controlled trial. *Clin. Obes.* 9:e12324. 10.1111/cob.12324 31172667

[B7] Bichler E. Spoendlin H. Rauchegger H. (1983). Degeneration of cochlear neurons after amikacin intoxication in the rat. *Arch. Otorhinolaryngol.* 237 201–208. 10.1007/bf00453725 6870653

[B8] Bourien J. Tang Y. Batrel C. Huet A. Lenoir M. Ladrech S. (2014). Contribution of auditory nerve fibers to compound action potential of the auditory nerve. *J. Neurophysiol.* 112 1025–1039. 10.1152/jn.00738.2013 24848461

[B9] Bozzo C. Sabbatini M. Tiberio R. Piffanelli V. Santoro C. Cannas M. (2006). Activation of caspase-8 triggers anoikis in human neuroblastoma cells. *Neurosci. Res.* 56 145–153. 10.1016/j.neures.2006.06.008 16872704

[B10] Brownell W. E. (1990). Outer hair cell electromotility and otoacoustic emissions. *Ear Hear.* 11 82–92. 10.1097/00003446-199004000-00003 2187727 PMC2796234

[B11] Brownell W. E. Jacob S. Hakizimana P. Ulfendahl M. Fridberger A. (2011). Membrane cholesterol modulates cochlear electromechanics. *Pflugers Arch.* 461 677–686. 10.1007/s00424-011-0942-5 21373862 PMC3098987

[B12] Chen G. D. Daszynski D. M. Ding D. Jiang H. Woolman T. Blessing K. (2020). Novel oral multifunctional antioxidant prevents noise-induced hearing loss and hair cell loss. *Hear. Res.* 388:107880. 10.1016/j.heares.2019.107880 31945692 PMC7039757

[B13] Chen H. Xing Y. Xia L. Chen Z. Yin S. Wang J. (2018). AAV-mediated NT-3 overexpression protects cochleae against noise-induced synaptopathy. *Gene Ther.* 25 251–259. 10.1038/s41434-018-0012-0 29535374 PMC6062503

[B14] Cheng A. G. Cunningham L. L. Rubel E. W. (2005). Mechanisms of hair cell death and protection. *Curr. Opin. Otolaryngol. Head Neck Surg.* 13 343–348. 10.1097/01.moo.0000186799.45377.63 16282762

[B15] Cho Y. Y. Kwon O. H. Park M. K. Kim T. W. Chung S. (2019). Elevated cellular cholesterol in familial Alzheimer’s presenilin 1 mutation is associated with lipid raft localization of beta-amyloid precursor protein. *PLoS One* 14:e0210535. 10.1371/journal.pone.0210535 30682043 PMC6347419

[B16] Coisne C. Tilloy S. Monflier E. Wils D. Fenart L. Gosselet F. (2016). Cyclodextrins as emerging therapeutic tools in the treatment of cholesterol-associated vascular and neurodegenerative diseases. *Molecules* 21:1748. 10.3390/molecules21121748 27999408 PMC6273856

[B17] Cronin S. Lin A. Thompson K. Hoenerhoff M. Duncan R. K. (2015). Hearing loss and otopathology following systemic and intracerebroventricular delivery of 2-hydroxypropyl-beta-cyclodextrin. *J. Assoc. Res. Otolaryngol.* 16 599–611. 10.1007/s10162-015-0528-6 26055150 PMC4569609

[B18] Crumling M. A. King K. A. Duncan R. K. (2017). Cyclodextrins and iatrogenic hearing loss: new drugs with significant risk. *Front. Cell. Neurosci.* 11:355. 10.3389/fncel.2017.00355 29163061 PMC5676048

[B19] Crumling M. A. Liu L. Thomas P. V. Benson J. Kanicki A. Kabara L. (2012). Hearing loss and hair cell death in mice given the cholesterol-chelating agent hydroxypropyl-beta-cyclodextrin. *PLoS One* 7:e53280. 10.1371/journal.pone.0053280 23285273 PMC3532434

[B20] Dallos P. Harris D. (1978). Properties of auditory nerve responses in absence of outer hair cells. *J. Neurophysiol.* 41 365–383. 10.1152/jn.1978.41.2.365 650272

[B21] Davis M. E. Brewster M. E. (2004). Cyclodextrin-based pharmaceutics: past, present and future. *Nat. Rev. Drug Discov.* 3 1023–1035. 10.1038/nrd1576 15573101

[B22] Dheen S. T. Kaur C. Ling E. A. (2007). Microglial activation and its implications in the brain diseases. *Curr. Med. Chem.* 14 1189–1197. 10.2174/092986707780597961 17504139

[B23] Dietschy J. M. Turley S. D. (2004). Thematic review series: brain lipids. cholesterol metabolism in the central nervous system during early development and in the mature animal. *J. Lipid Res.* 45 1375–1397. 10.1194/jlr.r400004-jlr200 15254070

[B24] Ding D. L. Wang J. Salvi R. Henderson D. Hu B. H. McFadden S. L. (1999). Selective loss of inner hair cells and type-I ganglion neurons in carboplatin-treated chinchillas. Mechanisms of damage and protection. *Ann. N. Y. Acad. Sci.* 884 152–170. 10.1111/j.1749-6632.1999.tb08640.x 10842592

[B25] Ding D. Jiang H. Salvi R. (2021). Cochlear spiral ganglion neuron degeneration following cyclodextrin-induced hearing loss. *Hear. Res.* 400:108125. 10.1016/j.heares.2020.108125 33302057

[B26] Ding D. Jiang H. Salvi R. J. (2010). Mechanisms of rapid sensory hair-cell death following co-administration of gentamicin and ethacrynic acid. *Hear. Res.* 259 16–23. 10.1016/j.heares.2009.08.008 19715747 PMC2814920

[B27] Ding D. Manohar S. Jiang H. Salvi R. (2020). Hydroxypropyl-beta-cyclodextrin causes massive damage to the developing auditory and vestibular system. *Hear. Res.* 396:108073. 10.1016/j.heares.2020.108073 32956992

[B28] Ding D. Wang J. Zheng X.-Y. Salvi R. J. (1998). Early damage of spiral ganglion caused by carboplatin in chinchilla. *J. Audiol. Speech Pathol.* 6 65–67.

[B29] Favre D. Sans A. (1991). Dedifferentiation phenomena after denervation of mammalian adult vestibular receptors. *Neuroreport* 2 501–504. 10.1097/00001756-199109000-00001 1751803

[B30] Fritzsch B. Tessarollo L. Coppola E. Reichardt L. F. (2004). Neurotrophins in the ear: their roles in sensory neuron survival and fiber guidance. *Prog. Brain Res.* 146 265–278. 10.1016/s0079-6123(03)46017-2 14699969

[B31] Fu Y. Ding D. Jiang H. Salvi R. (2012). Ouabain-induced cochlear degeneration in rat. *Neurotox. Res.* 22 158–169. 10.1007/s12640-012-9320-0 22476946 PMC3368987

[B32] Garofalo T. Misasi R. Mattei V. Giammarioli A. M. Malorni W. Pontieri G. M. (2003). Association of the death-inducing signaling complex with microdomains after triggering through CD95/Fas. Evidence for caspase-8-ganglioside interaction in T cells. *J. Biol. Chem.* 278 8309–8315. 10.1074/jbc.m207618200 12499380

[B33] Jensen-Smith H. Hallworth R. (2007). Lateral wall protein content mediates alterations in cochlear outer hair cell mechanics before and after hearing onset. *Cell Motil. Cytoskeleton* 64 705–717. 10.1002/cm.20217 17615570 PMC1992524

[B34] Johnson K. R. Tian C. Gagnon L. H. Jiang H. Ding D. Salvi R. (2017). Effects of Cdh23 single nucleotide substitutions on age-related hearing loss in C57BL/6 and 129S1/Sv mice and comparisons with congenic strains. *Sci. Rep.* 7:44450. 10.1038/srep44450 PMC5347380 28287619

[B35] Kamar R. I. Organ-Darling L. E. Raphael R. M. (2012). Membrane cholesterol strongly influences confined diffusion of prestin. *Biophys. J.* 103 1627–1636. 10.1016/j.bpj.2012.07.052 23083705 PMC3475345

[B36] Kiernan A. E. Cordes R. Kopan R. Gossler A. Gridley T. (2005). The Notch ligands DLL1 and JAG2 act synergistically to regulate hair cell development in the mammalian inner ear. *Development* 132 4353–4362. 10.1242/dev.02002 16141228

[B37] Kim H. Han J. Park J. H. (2020). Cyclodextrin polymer improves atherosclerosis therapy and reduces ototoxicity. *J. Control Release* 319 77–86. 10.1016/j.jconrel.2019.12.021 31843641

[B38] King K. A. Gordon-Salant S. Yanjanin N. Zalewski C. Houser A. Porter F. D. (2014). Auditory phenotype of Niemann-Pick disease, type C1. *Ear Hear.* 35 110–117. 10.1097/aud.0b013e3182a362b8 24225652 PMC3895917

[B39] Koitchev K. Guilhaume A. Cazals Y. Aran J. M. (1982). Spiral ganglion changes after massive aminoglycoside treatment in the guinea pig. Counts and ultrastructure. *Acta Otolaryngol.* 94 431–438. 10.3109/00016488209128931 6184939

[B40] Kolodny E. H. (2000). Niemann-Pick disease. *Curr. Opin. Hematol.* 7 48–52. 10608504 10.1097/00062752-200001000-00009

[B41] Lambert D. O’Neill C. A. Padfield P. J. (2007). Methyl-beta-cyclodextrin increases permeability of Caco-2 cell monolayers by displacing specific claudins from cholesterol rich domains associated with tight junctions. *Cell. Physiol. Biochem.* 20 495–506. 10.1159/000107533 17762176

[B42] Laza-Knoerr A. L. Gref R. Couvreur P. (2010). Cyclodextrins for drug delivery. *J. Drug Target* 18 645–656. 20497090 10.3109/10611861003622552

[B43] Leake P. A. Rebscher S. J. Dore C. Akil O. (2019). AAV-mediated neurotrophin gene therapy promotes improved survival of cochlear spiral ganglion neurons in neonatally deafened cats: comparison of AAV2-hBDNF and AAV5-hGDNF. *J. Assoc. Res. Otolaryngol.* 20 341–361. 10.1007/s10162-019-00723-5 31222416 PMC6646500

[B44] Lee J. N. Kim S. G. Lim J. Y. Dutta R. K. Kim S. J. Choe S. K. (2016). 3-Aminotriazole protects from CoCl2-induced ototoxicity by inhibiting the generation of reactive oxygen species and proinflammatory cytokines in mice. *Arch. Toxicol.* 90 781–791. 10.1007/s00204-015-1506-9 25820916

[B45] Legendre K. Safieddine S. Kussel-Andermann P. Petit C. El-Amraoui A. (2008). alphaII-betaV spectrin bridges the plasma membrane and cortical lattice in the lateral wall of the auditory outer hair cells. *J. Cell Sci.* 121(Pt 20) 3347–3356. 10.1242/jcs.028134 18796539

[B46] Liberman M. C. Gao J. He D. Z. Wu X. Jia S. Zuo J. (2002). Prestin is required for electromotility of the outer hair cell and for the cochlear amplifier. *Nature* 419 300–304. 10.1038/nature01059 12239568

[B47] Lin H. W. Furman A. C. Kujawa S. G. Liberman M. C. (2011). Primary neural degeneration in the Guinea pig cochlea after reversible noise-induced threshold shift. *J. Assoc. Res. Otolaryngol.* 12 605–616. 10.1007/s10162-011-0277-0 21688060 PMC3173555

[B48] Lirussi F. Beccarello A. Zanette G. Monte A. De Donadon V. Velussi M. (2002). Silybin-beta-cyclodextrin in the treatment of patients with diabetes mellitus and alcoholic liver disease. Efficacy study of a new preparation of an anti-oxidant agent. *Diabetes Nutr. Metab.* 15 222–231. 12416659

[B49] Liu B. Ramirez C. M. Miller A. M. Repa J. J. Turley S. D. Dietschy J. M. (2010). Cyclodextrin overcomes the transport defect in nearly every organ of NPC1 mice leading to excretion of sequestered cholesterol as bile acid. *J. Lipid Res.* 51 933–944. 10.1194/jlr.m000257 19965601 PMC2853461

[B50] Liu X. Ding D. Chen G. D. Li L. Jiang H. Salvi R. (2020). 2-hydroxypropyl-beta-cyclodextrin ototoxicity in adult rats: rapid onset and massive destruction of both inner and outer hair cells above a critical dose. *Neurotox. Res.* 38 808–823. 10.1007/s12640-020-00252-7 32607920 PMC7484207

[B51] Loftsson T. Brewster M. E. (2010). Pharmaceutical applications of cyclodextrins: basic science and product development. *J. Pharm. Pharmacol.* 62 1607–1621. 10.1111/j.2042-7158.2010.01030.x 21039545

[B52] Ma Y. Wise A. K. Shepherd R. K. Richardson R. T. (2019). New molecular therapies for the treatment of hearing loss. *Pharmacol. Ther.* 200 190–209. 10.1016/j.pharmthera.2019.05.003 31075354 PMC6626560

[B53] Maarup T. J. Chen A. H. Porter F. D. Farhat N. Y. Ory D. S. Sidhu R. (2015). Intrathecal 2-hydroxypropyl-beta-cyclodextrin in a single patient with Niemann-Pick C1. *Mol. Genet. Metab.* 116 75–79. 10.1016/j.ymgme.2015.07.001 26189084 PMC4633280

[B54] Marttin E. Verhoef J. C. Merkus F. W. (1998). Efficacy, safety and mechanism of cyclodextrins as absorption enhancers in nasal delivery of peptide and protein drugs. *J. Drug Target* 6 17–36. 10.3109/10611869808997878 9769018

[B55] McFadden S. L. Ding D. Burkard R. F. Jiang H. Reaume A. G. Flood D. G. (1999). Cu/Zn SOD deficiency potentiates hearing loss and cochlear pathology in aged 129,CD-1 mice. *J. Comp. Neurol.* 413 101–112. 10.1002/(sici)1096-9861(19991011)413:1<101::aid-cne7>3.0.co;2-l 10464373

[B56] McFadden S. L. Ding D. Jiang H. Salvi R. J. (2004). Time course of efferent fiber and spiral ganglion cell degeneration following complete hair cell loss in the chinchilla. *Brain Res.* 997 40–51. 10.1016/j.brainres.2003.10.031 14715148

[B57] Mitrofanova A. Molina J. Santos J. Varona Guzman J. Morales X. A. Ducasa G. M. (2018). Hydroxypropyl-beta-cyclodextrin protects from kidney disease in experimental Alport syndrome and focal segmental glomerulosclerosis. *Kidney Int.* 94 1151–1159. 10.1016/j.kint.2018.06.031 30301568 PMC6278936

[B58] Mueller K. L. Jacques B. E. Kelley M. W. (2002). Fibroblast growth factor signaling regulates pillar cell development in the organ of corti. *J. Neurosci.* 22 9368–9377. 10.1523/jneurosci.22-21-09368.2002 12417662 PMC6758064

[B59] Müller M. (1991). Frequency representation in the rat cochlea. *Hear. Res.* 51 247–254. 10.1016/0378-5955(91)90041-7 2032960

[B60] Newton J. Milstien S. Spiegel S. (2018). Niemann-Pick type C disease: the atypical sphingolipidosis. *Adv. Biol. Regul.* 70 82–88. 10.1016/j.jbior.2018.08.001 30205942 PMC6327306

[B61] Nguyen T. V. Brownell W. E. (1998). Contribution of membrane cholesterol to outer hair cell lateral wall stiffness. *Otolaryngol. Head Neck Surg.* 119 14–20. 10.1016/s0194-5998(98)70167-6 9674509

[B62] Oesterle E. C. Campbell S. (2009). Supporting cell characteristics in long-deafened aged mouse ears. *J. Assoc. Res. Otolaryngol.* 10 525–544. 10.1007/s10162-009-0183-x 19644644 PMC2774416

[B63] Okamura W. H. Zhu G. D. Hill D. K. Thomas R. J. Ringe K. Borchardt D. B. (2002). Synthesis and NMR studies of (13)C-labeled vitamin D metabolites. *J. Org. Chem.* 67 1637–1650. 11871897 10.1021/jo011096y

[B64] Ory D. S. (2000). Niemann-Pick type C: a disorder of cellular cholesterol trafficking. *Biochim. Biophys. Acta* 1529 331–339. 10.1016/s1388-1981(00)00158-x 11111100

[B65] Otero-Espinar F. J. Luzardo-Alvarez A. Blanco-Mendez J. (2010). Cyclodextrins: more than pharmaceutical excipients. *Mini Rev. Med. Chem.* 10 715–725. 10.2174/138955710791572479 20482501

[B66] Park E. K. Park M. J. Lee S. H. Li Y. C. Kim J. Lee J. S. (2009). Cholesterol depletion induces anoikis-like apoptosis via FAK down-regulation and caveolae internalization. *J. Pathol.* 218 337–349. 10.1002/path.2531 19288501

[B67] Pickles J. O. Chir B. (2002). Roles of fibroblast growth factors in the inner ear. *Audiol. Neurootol.* 7 36–39. 10.1159/000046861 11914524

[B68] Pontikis C. C. Davidson C. D. Walkley S. U. Platt F. M. Begley D. J. (2013). Cyclodextrin alleviates neuronal storage of cholesterol in Niemann-Pick C disease without evidence of detectable blood-brain barrier permeability. *J. Inherit. Metab. Dis.* 36 491–498. 10.1007/s10545-012-9583-x 23412751 PMC3929395

[B69] Rajagopalan L. Greeson J. N. Xia A. Liu H. Sturm A. Raphael R. M. (2007). Tuning of the outer hair cell motor by membrane cholesterol. *J. Biol. Chem.* 282 36659–36670. 10.1074/jbc.m705078200 17933870 PMC2679373

[B70] Ramirez C. M. Liu B. Aqul A. Taylor A. M. Repa J. J. Turley S. D. (2011). Quantitative role of LAL, NPC2, and NPC1 in lysosomal cholesterol processing defined by genetic and pharmacological manipulations. *J. Lipid Res.* 52 688–698. 10.1194/jlr.m013789 21289032 PMC3284162

[B71] Ramirez C. M. Liu B. Taylor A. M. Repa J. J. Burns D. K. Weinberg A. G. (2010). Weekly cyclodextrin administration normalizes cholesterol metabolism in nearly every organ of the Niemann-Pick type C1 mouse and markedly prolongs life. *Pediatr. Res.* 68 309–315. 10.1203/pdr.0b013e3181ee4dd2 20581737 PMC3065173

[B72] Ren L. L. Wu Y. Han D. Zhao L. D. Sun Q. M. Guo W. W. (2010). Math1 gene transfer based on the delivery system of quaternized chitosan/Na-carboxymethyl-beta-cyclodextrin nanoparticles. *J. Nanosci. Nanotechnol.* 10 7262–7265. 10.1166/jnn.2010.2822 21137911

[B73] Rybak L. P. Whitworth C. A. (2005). Ototoxicity: therapeutic opportunities. *Drug Discov. Today* 10 1313–1321. 10.1016/s1359-6446(05)03552-x 16214676

[B74] Salvesen G. S. (2002). Caspases and apoptosis. *Essays Biochem.* 38 9–19. 12463158 10.1042/bse0380009

[B75] Schonfelder U. Radestock A. Elsner P. Hipler U. C. (2006). Cyclodextrin-induced apoptosis in human keratinocytes is caspase-8 dependent and accompanied by mitochondrial cytochrome c release. *Exp. Dermatol.* 15 883–890. 10.1111/j.1600-0625.2006.00481.x 17002685

[B76] Stella V. J. He Q. (2008). Cyclodextrins. *Toxicol. Pathol.* 36 30–42. 18337219 10.1177/0192623307310945

[B77] Takahashi S. Homma K. Zhou Y. Nishimura S. Duan C. Chen J. (2016). Susceptibility of outer hair cells to cholesterol chelator 2-hydroxypropyl-beta-cyclodextrine is prestin-dependent. *Sci. Rep.* 6:21973. 10.1038/srep21973 PMC4763217 26903308

[B78] Tang G. P. Guo H. Y. Alexis F. Wang X. Zeng S. Lim T. M. (2006). Low molecular weight polyethylenimines linked by beta-cyclodextrin for gene transfer into the nervous system. *J. Gene Med.* 8 736–744. 10.1002/jgm.874 16550629

[B79] Taylor A. M. Liu B. Mari Y. Liu B. Repa J. J. (2012). Cyclodextrin mediates rapid changes in lipid balance in Npc1-/- mice without carrying cholesterol through the bloodstream. *J. Lipid Res.* 53 2331–2342. 10.1194/jlr.m028241 22892156 PMC3466002

[B80] Taylor R. R. Jagger D. J. Forge A. (2012). Defining the cellular environment in the organ of Corti following extensive hair cell loss: a basis for future sensory cell replacement in the Cochlea. *PLoS One* 7:e30577. 10.1371/journal.pone.0030577 22299045 PMC3267727

[B81] Tillinger J. A. Gupta C. Ila K. Ahmed J. Mittal J. Van De Water T.R. (2018). l-N-acetylcysteine protects outer hair cells against TNFalpha initiated ototoxicity in vitro. *Acta Otolaryngol.* 138 676–684. 10.1080/00016489.2018.1440086 29513056

[B82] Uchenna Agu R. Jorissen M. Willems T. Van den Mooter G. Kinget R. Verbeke N. (2000). Safety assessment of selected cyclodextrins – effect on ciliary activity using a human cell suspension culture model exhibiting in vitro ciliogenesis. *Int. J. Pharm.* 193 219–226. 10.1016/s0378-5173(99)00342-7 10606785

[B83] Vecsernyes M. Fenyvesi F. Bacskay I. Deli M. A. Szente L. Fenyvesi E. (2014). Cyclodextrins, blood-brain barrier, and treatment of neurological diseases. *Arch. Med. Res.* 45 711–729. 10.1016/j.arcmed.2014.11.020 25482528

[B84] Walenbergh S. M. Houben T. Hendrikx T. Jeurissen M. L. van Gorp P. J. Vaes N. (2015). Weekly treatment of 2-hydroxypropyl-beta-cyclodextrin improves intracellular cholesterol levels in LDL receptor knockout mice. *Int. J. Mol. Sci.* 16 21056–21069. 10.3390/ijms160921056 26404254 PMC4613241

[B85] Wang J. Ding D. Salvi R. J. (2003). Carboplatin-induced early cochlear lesion in chinchillas. *Hear. Res.* 181 65–72. 10.1016/s0378-5955(03)00176-x 12855364

[B86] Wang J. Ladrech S. Pujol R. Brabet P. Van De Water T. R. Puel J. L. (2004). Caspase inhibitors, but not c-Jun NH2-terminal kinase inhibitor treatment, prevent cisplatin-induced hearing loss. *Cancer Res.* 64 9217–9224. 10.1158/0008-5472.can-04-1581 15604295

[B87] Ward S. O’Donnell P. Fernandez S. Vite C. H. (2010). 2-hydroxypropyl-beta-cyclodextrin raises hearing threshold in normal cats and in cats with Niemann-Pick type C disease. *Pediatr. Res.* 68 52–56. 10.1203/pdr.0b013e3181df4623 20357695 PMC2913583

[B88] Wood M. B. Zuo J. (2017). The contribution of immune infiltrates to ototoxicity and cochlear hair cell loss. *Front. Cell. Neurosci.* 11:106. 10.3389/fncel.2017.00106 28446866 PMC5388681

[B89] Wu N. Li M. Chen Z. T. Zhang X. B. Liu H. Z. Li Z. (2013). In vivo delivery of Atoh1 gene to rat cochlea using a dendrimer-based nanocarrier. *J. Biomed. Nanotechnol.* 9 1736–1745. 10.1166/jbn.2013.1684 24015503

[B90] Yagi M. Kanzaki S. Kawamoto K. Shin B. Shah P. P. Magal E. (2000). Spiral ganglion neurons are protected from degeneration by GDNF gene therapy. *J. Assoc. Res. Otolaryngol.* 1 315–325. 11547811 10.1007/s101620010011 PMC2957193

[B91] Zhang J. Sun H. Salvi R. Ding D. (2018). Paraquat initially damages cochlear support cells leading to anoikis-like hair cell death. *Hear. Res.* 364 129–141. 10.1016/j.heares.2018.03.014 29563067 PMC5984146

[B92] Zhou Y. Takahashi S. Homma K. Duan C. Zheng J. Cheatham M. A. (2018). The susceptibility of cochlear outer hair cells to cyclodextrin is not related to their electromotile activity. *Acta Neuropathol. Commun.* 6:98. 10.1186/s40478-018-0599-9 PMC6151916 30249300

[B93] Zimmer S. Grebe A. Bakke S. S. Bode N. Halvorsen B. Ulas T. (2016). Cyclodextrin promotes atherosclerosis regression via macrophage reprogramming. *Sci. Transl. Med.* 8:333ra50. 10.1126/scitranslmed.aad6100 27053774 PMC4878149

